# Green Synthesis of Silver Nanoparticles From Various Leaves Extract: Antioxidant, Anti‐Inflammatory, Antibacterial Activities and Inhibitory Effect on Glutathione S‐Transferase

**DOI:** 10.1002/fsn3.72288

**Published:** 2026-09-15

**Authors:** Zuhal Sahin, Nourhane A. Darwich, Tulay Duran, Davut Avci, Luís R. Silva, Fatih Sonmez, Mahmoud I. Khalil

**Affiliations:** ^1^ Pamukova Vocational School Sakarya University of Applied Sciences Sakarya Turkey; ^2^ Department of Biological Sciences, Faculty of Science Beirut Arab University Beirut Lebanon; ^3^ Department of Physics, Faculty of Science Sakarya University Sakarya Turkey; ^4^ SPRINT—IPG, Sport Physical Activity and Health Research e Innovation Center Polytechnic Institute of Guarda Guarda Portugal; ^5^ RISE‐Health—Faculdade de Ciências da Saúde, Universidade da Beira Interior Health Sciences Research Centre University of Beira Interior Covilhã Portugal; ^6^ CERES—UC, Department of Chemical Engineering University of Coimbra Coimbra Portugal; ^7^ Molecular Biology Unit, Department of Zoology, Faculty of Science Alexandria University Alexandria Egypt

**Keywords:** antibacterial, anti‐inflammatory, antioxidant, GST enzyme inhibition, leaves extracts, nanoparticles

## Abstract

Nanoparticles have attracted considerable attention due to their high surface area‐to‐volume ratio, tunable size, unique physicochemical properties, and, depending on their composition, favorable electrical, photocatalytic, and biocompatible characteristics. Silver nanoparticles (AgNPs) were prepared using a green synthesis approach employing the leaf extracts of *Cydonia oblonga* , *Actinidia deliciosa* , *Prunus spinosa* , and *Prunus laurocerasus* . The synthesized AgNPs were characterized using UV‐Vis spectroscopy, FTIR, and SEM‐EDX. Furthermore, the potential antioxidant, anti‐inflammatory, antibacterial, and GST inhibitory activities of these AgNPs were assessed. Among all prepared nanomaterials, AgNPs produced using *C. oblonga* leaf extract (AgNP‐1) displayed the highest antioxidant capacity (IC_50_ = 4.14 ± 0.04 μg/mL) and outstanding anti‐inflammatory effects (98.48%). Additionally, AgNP‐1 showed the strongest GST inhibition activity (IC50 = 1.306 ± 0.12 mg/mL). Conversely, AgNPs produced using *P. laurocerasus* leaf extract (AgNP‐4) exhibited the strongest antibacterial activity, displaying MIC values ranging from 39 to 78 μg/mL against different bacterial species, and the greatest GST inhibitory activity (IC_50_ = 0.965 ± 0.02 mg/mL). Moreover, molecular docking studies showed that the phenolic hydroxyl functional groups of the metabolites found in the plant extracts exhibited strong interaction with the GST active site through conventional hydrogen bond formation, electrostatic interactions, hydrophobic interactions, and metal acceptor interactions, whereas AgNPs interacted with GST primarily through metal acceptor interactions. Therefore, the current study revealed that plant‐mediated, biologically synthesized AgNPs exhibit promising biomedical properties and may serve as potential candidates for the development of antimicrobial agents, anti‐inflammatory agents, antioxidants, and enzyme inhibitors because of their significant antibacterial, anti‐inflammatory, antioxidant, and GST‐inhibitory effects.

## Graphical abstract

Green‐synthesized silver nanoparticles (AgNPs) were prepared using plant leaf extract and evaluated for their biological activities. The synthesized AgNPs demonstrated antibacterial, antioxidant, anti‐inflammatory, and glutathione S‐transferase (GST) inhibitory activities, highlighting their potential as multifunctional bioactive nanomaterials for food, health, and biomedical applications.

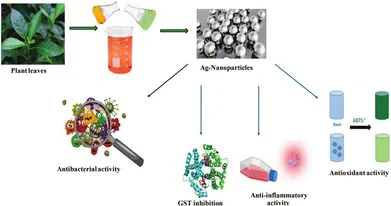

## 1 Introduction

**FIGURE 1:**
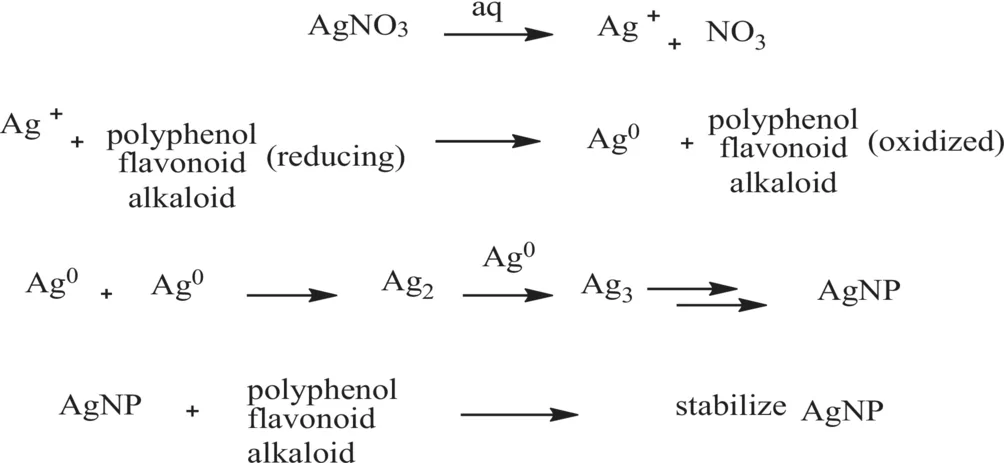
Mechanism of AgNO_3_ reduction into AgNPs.

Nanotechnology is mostly used in the automotive, electronics, pharmaceutical, textile, cosmetic, food, and agricultural sectors. This technique entails the use of particles that are 100 nm or less in size, which are generally called nanoparticles (Wang et al. 2011; Asefian and Ghavam 2024; Pitkethly 2024). Nanoparticles can be synthesized from a variety of materials, including various metals, such as copper, zinc, titanium, magnesium, gold, alginate, and silver, as well as polymeric and biopolymeric materials such as alginate (Akyol et al. 2024). Silver nanoparticles (AgNPs) are synthesized using many processes, including physical, chemical, photochemical, microemulsion, biological, and microwave‐assisted methods (Joseph and Mathew 2014). While physical procedures are expensive, chemical methods have been associated with negative consequences due to the adsorption of some harmful compounds on the surface (Khatoon et al. 2017). There are also environmentally friendly, highly efficient, and cost‐effective synthesis methods. One of these is biological synthesis, which uses microorganisms, enzymes, plants, or plant extracts as reducing and stabilizing agents (Patel et al. 2023). In biological synthesis, glycosides, terpenoids, alkaloids, phenolic compounds, flavonoids, and other plant components are used as reducing agents for the reduction of Ag^+^ ions derived from silver nitrate (AgNO_3_) (Elangovan et al. 2022; Khane et al. 2022). The functional groups (—OH and —COOH) in these molecules interact with Ag^+^ ions. Plant‐derived phytochemicals containing hydroxyl and carboxyl functional groups play important roles in the reduction of Ag^+^ ions to metallic silver (Ag^0^) and the stabilization of the synthesized AgNPs (Figure 1).

AgNPs are particularly significant among metal nanoparticles because of their beneficial properties, such as conductivity, stability, and antimicrobial activity (Abdelghany et al. 2018). AgNPs possess unique physicochemical and biological properties that support their wide range of biomedical and pharmaceutical applications (Fahim et al. 2024). Green‐synthesized AgNPs play an important role in biomedical and pharmaceutical applications, as well as other fields such as environmental remediation, agriculture, food, renewable energy, textiles, and medical sciences. Biologically synthesized AgNPs are preferred for medical applications due to their greater biocompatibility as compared with chemically and physically synthesized AgNPs (Abdelghany et al. 2018). Green synthesis aims to employ renewable resources such as plant extracts and microorganisms, reduce waste generation, and use nontoxic chemicals and solvents such as water. The plant extracts utilized in green synthesis function as both reducing and stabilizing agents, thereby enhancing the biocompatibility and biological activity of the generated nanoparticles (Moradi et al. 2021; Dua et al. 2023; Eker et al. 2025). Given the significance of AgNPs synthesis, the main objective of this study was to develop a green synthesis method for preparing AgNPs from *Cydonia oblonga* (AgNP‐1), *Actinidia deliciosa* (AgNP‐2), *Prunus spinosa* (AgNP‐3), and *P. laurocerasus* (AgNP‐4) leaves, which are rich in bioactive compounds.

Nevertheless, even though there is extensive literature regarding the biosynthesis of AgNPs using plants, comparative studies evaluating AgNPs synthesized from different medicinal plants under similar experimental conditions remain limited. Moreover, the majority of studies focus only on individual biological activities, with few investigations integrating antioxidant activity, antibacterial activity, anti‐inflammatory potential, enzyme inhibitory activity, and molecular docking analysis into a comprehensive evaluation. Therefore, a systematic comparative assessment of the biological activities and molecular interactions of plant‐mediated AgNPs synthesized under standardized conditions is still lacking. The present study aims to develop a sustainable method for the green synthesis of AgNPs by using aqueous extracts of *C. oblonga* (AgNP‐1), *A. deliciosa* (AgNP‐2), *P. spinosa* (AgNP‐3), and *P. laurocerasus* (AgNP‐4). The synthesized AgNPs were characterized by UV‐Vis spectroscopy, FTIR spectroscopy, and SEM‐EDX analysis. Additionally, the antioxidant properties, antibacterial activity, anti‐inflammatory potential, and GST inhibitory activity of the prepared AgNPs were evaluated. Furthermore, a molecular docking study was performed to examine the interaction between selected bioactive compounds identified in the plant extracts and the GST enzyme.

## 2 Materials and Experimental Procedures

### 2.1 Reagents and Chemical Substances

All chemical agents and compounds utilized throughout this investigation were supplied by Sigma‐Aldrich and possessed analytical‐grade quality with a purity exceeding 99%. Glutathione S‐transferase from equine liver (EC Number: 2.5.1.18) was used for the enzyme inhibition assay. UV‐Vis spectral analysis was performed using a Shimadzu UV‐visible spectrophotometer (UVmini‐1240, Shimadzu, Japan) within the wavelength range of 300–800 nm. FTIR analysis of the extracts and synthesized AgNPs was carried out over the spectral range of 4000–400/cm using a Perkin Elmer UATR‐TWO spectrophotometer (Perkin Elmer Spectrum‐Two equipped with ATR). Scanning electron microscopy coupled with energy‐dispersive X‐ray spectroscopy (SEM‐EDX; Thermo Scientific Axia ChemiSEM) was employed to investigate the surface morphology and elemental composition of the synthesized AgNPs.

### 2.2 Plant Material Collection and Extract Preparation

**FIGURE 2:**
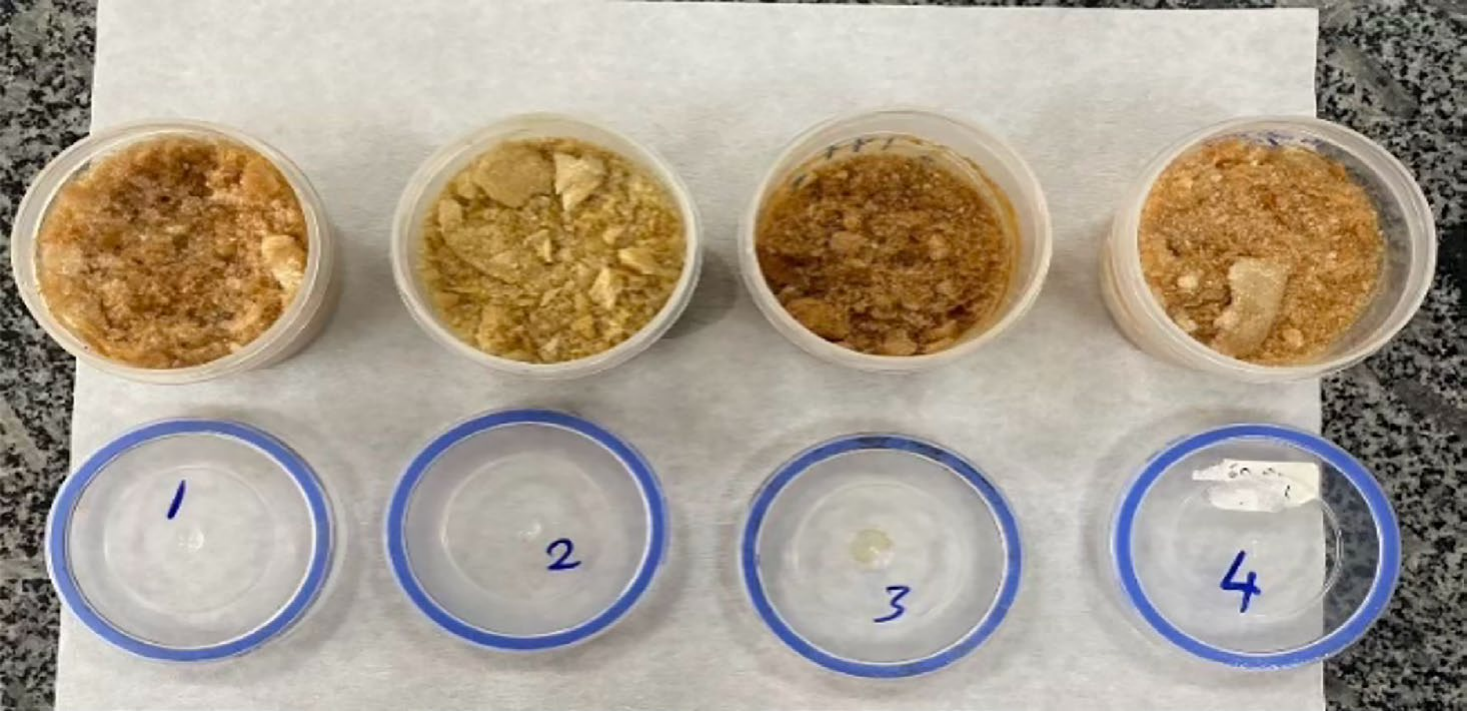
Powdered filtrate of leaves after lyophilization (1) *Cydonia oblonga* ; (2) *Actinidia deliciosa* ; (3) *Prunus spinosa* ; (4) *Prunus laurocerasus* .

*C. oblonga* , *A. deliciosa* , *Prunus spinosa* , and *P. laurocerasus* leaves were collected from a location at latitude 40°29′1.6764″ N and longitude 30°10′58.0800″ E, at an altitude of 80 m above sea level in Sakarya, Turkey. The collected leaves were immediately transported to the laboratory and dried in an oven at 30°C. The dried plant materials were powdered to obtain a particle size of less than 910 μm and subsequently used for extraction. Dry leaf samples were crushed in a Waring blender with distilled water at a 1:10 (w/v) ratio (1 g plant material to 10 mL distilled water). The resulting mixture was filtered for 2 h, followed by filtration through Whatman No. 1 filter paper under vacuum. The resulting solution was subjected to freeze‐drying at −45°C and a reduced pressure of 0.045 mbar for 3 days in a lyophilizer (Labconco Freezone 6.0, Labconco, USA) (Figure 2).

### 2.3 Green Synthesis of AgNPs

**FIGURE 3:**
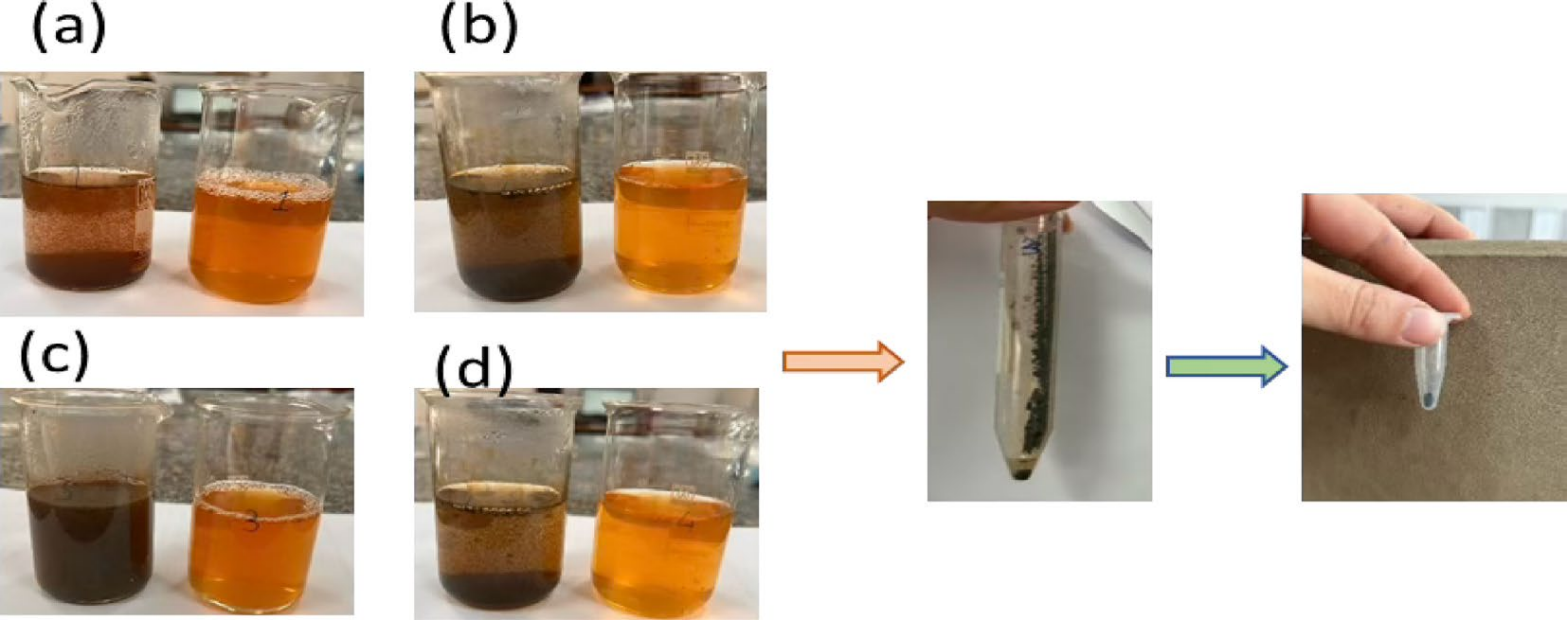
Leaf extracts before and after nanosilver synthesis. (a) *Cydonia oblonga* (Extract‐1, AgNP‐1), (b) *Actinidia deliciosa* (Extract‐2, AgNP‐2), (c) *Prunus spinosa* (Extract‐3, AgNP‐3), and (d) *Prunus laurocerasus* (Extract‐4, AgNP‐4).

A total of 500 mg of each powdered extract was dissolved in 100 mL of distilled water. Subsequently, 10 mL of the prepared extract solution was mixed with 90 mL of aqueous AgNO_3_ solution (0.1 M) and stirred at 60°C–80°C for 1 h. The formation of AgNPs was indicated by the gradual appearance of a dark brown color. Nanoparticle formation was monitored every 15 min using UV‐Vis spectroscopy, and the synthesis process was completed after 1 h. The extract‐AgNO_3_ mixture was centrifuged at 5000 × *g* for 15 min, and the resulting pellet was separated from the supernatant. The obtained pellet was washed thoroughly with distilled water three times, followed by repeated centrifugation to remove residual impurities. The purified pellet was dried at 45°C for 72 h (Dua et al. 2023) (Figure 3).

### 2.4 Phytochemical Properties

#### 2.4.1 Analysis of Total Phenolic Content (TPC)

TPC was analyzed using the Folin–Ciocalteu method (Guldane 2025). Folin–Ciocalteu reagent was used for TPC analysis. Aqueous samples of AgNPs‐1–4 (1000 μg/mL) were prepared. Subsequently, 100 μL of the samples or gallic acid standard solutions (62.5–1000 mg/L) were added to a solution composed of 2 mL of distilled water and 0.2 mL of Folin–Ciocalteu reagent. Then, the reaction mixture was incubated for 3 min at room temperature, followed by the addition of 1 mL of sodium carbonate solution (25%, w/v). The reaction mixtures were kept in the dark at room temperature for 1 h. Absorbance was measured at a wavelength of 765 nm using a UV‐Visible Spectrophotometer. Gallic acid served as the reference standard. The equation of the calibration curve of gallic acid was *y* = 0.0022*x* + 0.2006 (*R* ^2^ = 0.9972). The TPC was expressed as mg GAE/g.

#### 2.4.2 Evaluation of Total Flavonoid Content (TFC)

The total flavonoid content (TFC) was analyzed using a previously reported method (Elshazly et al. 2023). The assay was performed by preparing a solution composed of 5 mL of distilled water and 1 mL of AgNP. A solution was prepared from 5 mL of distilled water and 1 mL of AgNP‐1–4 aqueous solution (1 mg/mL). Following the addition of 0.3 mL of NaNO_2_ solution (5%, w/v) and 0.6 mL of AlCl_3_ solution (10%, w/v), the mixture was left at room temperature for 5 min. Afterwards, 2 mL of 1 M NaOH solution was added, and the volume was made up to 10 mL with distilled water. Absorbance was measured at 510 nm against a distilled water blank using a UV‐Vis spectrophotometer. Total flavonoid content was expressed as mg catechin equivalents (CE)/g sample.

### 2.5 Antioxidant Assays

#### 2.5.1 DPPH Radical Scavenging Activity

The antioxidant potential of the samples was estimated based on their ability of the sample to decolorize the DPPH (2,2‐diphenyl‐1‐picrylhydrazyl) solution, which initially appears purple in color (Alam et al. 2025). The DPPH test consisted of mixing 3 mL of DPPH solution (5 mg of DPPH dissolved in 250 mL methanol) with 0.5 mL of the sample, at concentrations ranging from. The mixtures were incubated in the dark for 30 min, after which their absorbance was measured using a UV‐Vis spectrophotometer at 517 nm. The antioxidant activity was expressed as percentage inhibition (%I) at each concentration. The baseline absorbance due to the intrinsic surface plasmon resonance (SPR) of AgNPs was subtracted using nanoparticle blank controls containing the corresponding AgNP samples without DPPH.

$$\%I=1-\frac{A\;\text{sample}}{A\;\text{control}}*100$$

#### 2.5.2 ABTS Radical Scavenging Analysis

The antioxidant activity of the synthesized silver nanoparticles against ABTS [2,2′‐azino‐bis(3‐ethylbenzothiazoline‐6‐sulfonic acid)] was determined according to the method described by (Karan, Gonulalan, et al. 2024; Karan, Erenler, et al. 2024). The ABTS^˙+^ radical cation was produced by oxidizing ABTS with potassium persulfate. ABTS diammonium salt (7 mM) was mixed with potassium persulfate and incubated in the dark for 24 h. The resulting solution was diluted with distilled water to obtain an absorbance of 0.70 ± 0.02 by dilution with distilled water. Different concentrations of the sample solutions (31.25–1000 μg/mL) (0.2 mL) were added to the diluted ABTS solution (3 mL), and the absorbance was measured at 734 nm after 6 min of incubation, using a UV‐Vis spectrophotometer. The diluted ABTS^˙+^ solution without sample served as the blank. Results were reported as IC_50_ values (μg/mL). The corresponding nanoparticle blanks (without ABTS) were systematically subtracted to eliminate optical interference caused by the surface plasmon resonance (SPR) band of the nanoparticles.

### 2.6 Anti‐Inflammatory (Bovine Serum Albumin Denaturation) Assay

The anti‐inflammatory effect of the synthesized AgNPs was studied by evaluating bovine serum albumin (BSA) protein denaturation (Sabarathinam and Madhulaxmi 2021). The concentration of AgNPs used was 1000 μg/mL. Diclofenac sodium was used as the reference compound at the same concentration. The test solution (500 μL) consisted of 450 μL of BSA solution (5% w/v) and 50 μL of either leaf extract solution or AgNPs, whereas the control solution (500 μL) contained 450 μL of BSA solution and 50 μL of distilled water. The product control solution (500 μL) contained 450 μL of distilled water and 50 μL of leaf extract solution or AgNPs solution. The standard solution (500 μL) consisted of 450 μL of BSA solution and 50 μL of diclofenac sodium solution. All the prepared solutions were adjusted to pH 6.3 with 1 N HCl. Samples were heated in a water bath for 20 min at 37°C and subsequently for 5 min at 57°C. Phosphate buffer solution (pH 6.3; 2.5 mL) was added to the samples, which were then allowed to cool. Absorbance was measured using a UV‐Vis spectrophotometer at 660 nm. Percentage inhibition of protein denaturation was calculated using the following equation:

$$\%I=100-\left(\frac{A\;\text{test solution}-A\;\text{product control}}{A\;\text{test control}}*100\right)$$

### 2.7 Assessment of Glutathione S‐Transferase Inhibition

The GST inhibition experiment was carried out using a reaction mixture prepared in a cuvette, which consisted of 100 μL of 20 mM glutathione and 150 μL of 25 mM CDNB (1‐chloro‐2,4‐dinitrobenzene) solution. Additionally, 2500 μL of 100 mM potassium phosphate buffer (pH 6.5) and 200 μL of GST enzyme isolated from horse liver were added to the mixture. After that, 200 μL of AgNP solution (1000 μg/mL) was incorporated into the reaction mixture. The absorbance was measured at 340 nm, initially (0 min) and subsequently at 1‐min intervals using a UV‐Vis spectrophotometer (Bhavi et al. 2024). Similarly, blank samples, which contained all reaction components except glutathione and AgNPs, were prepared. The percentage inhibition was determined based on the absorbance data obtained, and the results were expressed as IC_50_ (mg/mL).

### 2.8 Antibacterial Activity Assay

The antibacterial activity test involved the use of seven bacterial strains obtained from the ATCC collections. These included Gram‐negative bacteria such as *Escherichia coli* ATCC 25922, *Salmonella enteritidis* ATCC 13076, *Salmonella typhimurium* ATCC 14028, and *Pseudomonas aeruginosa* ATCC 9027, along with three Gram‐positive strains ( *Bacillus cereus* ATCC 11778, *Listeria monocytogenes* ATCC 7644, and *Staphylococcus aureus* ATCC 29213). All bacterial strains were obtained from the culture collection of the Microbiology Laboratory at the Pamukova Vocational School of Sakarya University of Applied Sciences, located in Sakarya, Turkey. These bacteria were preserved in broth medium containing 20% glycerol at −70°C. They were cultured on Mueller–Hinton agar (MHA) before conducting the experiments to ensure optimal growth conditions. The plant extracts and AgNPs for antimicrobial testing were dissolved in 5% DMSO to obtain a final concentration of 5000 μg/mL, followed by filtration through a sterile 0.45‐μm membrane filter (Bhavi et al. 2024).

#### 2.8.1 Disk Diffusion Method

The disk diffusion method for testing susceptibility was conducted following the CLSI guidelines with minor modifications (CLSI 2024a). A culture of a single colony of Gram‐positive and Gram‐negative bacteria was inoculated into tryptic soy broth (TSB) medium. This was done to achieve a bacterial suspension equivalent to the 0.5 McFarland standard (OD_625_ nm). The culture was then evenly spread on Mueller–Hinton agar (MHA) plates. Then, sterile filter paper disks (6 mm in diameter), soaked in 20 μL of the plant extract and AgNP suspensions, were placed onto the inoculated MHA agar plates. The plates were incubated at 35°C for 24 h. Ampicillin disks (6 mm in diameter) served as the positive control, whereas 5% DMSO served as the negative control. Each experiment was performed in triplicate.

#### 2.8.2 Evaluation of Minimum Inhibitory and Bactericidal Concentrations

The minimum inhibitory concentration (MIC) values were determined using the broth microdilution method according to the CLSI guidelines in 96‐well microplates (CLSI 2024b). The bacterial cultures were adjusted to the 0.5 McFarland turbidity standard and diluted to obtain a final inoculum concentration of 5 × 10^5^ CFU/mL in 0.85% NaCl solution. Serial twofold dilutions of the extracts or AgNPs were prepared in cation‐adjusted Mueller–Hinton broth (MHB) at concentrations ranging from 5000 to 9.8 μg/mL. The initial concentration of each tested sample was 5000 μg/mL (5 mg/mL).

Briefly, 100 μL of bacterial culture was added to each well containing 100 μL of the extract or AgNPs in MHB medium. The plates were incubated at 35°C for 16–20 h for both Gram‐positive and Gram‐negative bacteria. The lowest concentration of either the extract or AgNPs showing no visible bacterial growth in the microplate wells was considered the MIC. All experiments were performed in duplicate and independently repeated three times. The minimum bactericidal concentration (MBC) values of the extracts and AgNPs were also determined. Following incubation, 20‐μL aliquots from wells showing no visible growth were spread onto Mueller–Hinton agar (MHA) plates and incubated at 37°C for 18–24 h. The MBC value was defined as the lowest concentration showing complete (100%) bacterial growth inhibition.

### 2.9 Molecular Docking Assay

Docking studies were conducted using AutoDock4 (Morris et al. 2009) software using the graphical user interface AutoDockTools (ADT version 1.5.7) (Sanner 1999) to explore the interactions of the target protein with the major phytoconstituents found in the extracts, such as 5‐*O*‐caffeoylquinic acid in Extract‐1 (Oliveira et al. 2012), quercetin in Extract‐2 (Lv et al. 2021), cyanidin in Extract‐3 (Velickovic et al. 2021), and chlorogenic acid in Extract‐4 (Karabegovic et al. 2014). In addition, AgNPs were modeled based on the silver crystalline structure (Kadir et al. 2024). The crystal structure of glutathione S‐transferase (GST) (PDB code: 7BIB) was retrieved from the Protein Data Bank and served as the target protein. The three‐dimensional structures of the phytochemicals and AgNPs were prepared in PDB format. The program AutoDockTools (version 1.5.7) was applied to assign Kollman charges, calculate the degrees of rotational freedom through the identification of rotatable bonds, and combine nonpolar hydrogens with the appropriate carbon atoms. The docking analysis between the flexible structures of the phytochemicals, AgNPs, and the target protein was carried out using the Lamarckian Genetic Algorithm (LGA) (Morris et al. 1998). The PDBQT‐formatted receptor and ligand files (target.pdbqt and ligand.pdbqt), as well as the grid parameter file, were generated using AutoDock 4.2, whereas AutoGrid 4 was employed to define the docking region. To accommodate the binding site and allow optimal ligand mobility, the grid box parameters were adjusted accordingly. To evaluate the binding interactions, a 3D affinity grid with a spacing of 1.0 Å was calculated at the binding site of the phytochemicals and AgNPs. Atom types considered for the receptor included A, C, HD, N, OA, and SA, whereas the ligand atom types included A, C, HD, and OA. The Ag atom was specifically included for the AgNPs, along with electrostatic (e) and desolvation (d) parameters. The docking simulations were performed using the following parameters: population size = 150, mutation rate = 0.02, elitism = 1, crossover rate = 0.8, and local search rate = 0.06. The maximum number of energy calculations was set to 2,500,000. The optimal docking poses were selected on the basis of an RMSD cut‐off of 2.0 Å. The docking protocol was validated through a redocking procedure in which the native ligand was reintroduced into the binding site. The optimal binding pose yielded an RMSD of 1.48 Å, demonstrating accurate reproduction of the experimentally observed binding conformation.

For Extract‐1, a grid box comprising 80 × 60 × 68 points along the *x*, *y*, and *z* axes was constructed, centered at coordinates 22.989 (*x*), −0.116 (*y*), and 10.778 (*z*). For Extract‐2, a grid box comprising 72 × 68 × 66 points along the *x*, *y*, and *z* axes was constructed, centered at coordinates 22.989 (*x*), −0.116 (*y*), and 10.778 (*z*). For Extract‐3, a grid box comprising 70 × 60 × 62 points along the *x*, *y*, and *z* axes was constructed, centered at coordinates 22.989 (*x*), −0.116 (*y*), and 10.778 (*z*). For Extract‐4, a grid box comprising 72 × 60 × 62 points along the *x*, *y*, and *z* axes was constructed, centered at coordinates 22.989 (*x*), −0.116 (*y*), and 10.778 (*z*). For AgNPs, a grid box comprising 68 × 66 × 66 points along the *x*, *y*, and *z* axes was constructed, centered at coordinates 22.989 (*x*), −0.116 (*y*), and 10.778 (*z*). The obtained docking data were interpreted to assess the comparative binding strengths and stability profiles of the ligand–protein complexes. Additionally, the interactions of the phytochemicals/AgNPs with the amino acid residues present in GST were visualized using two‐dimensional (2D) and three‐dimensional (3D) images generated with Discovery Studio 4.0 (Dassault Systemes Biovia 2016).

### 2.10 Statistical Analysis

All experiments were performed in triplicate, and the results are expressed as the mean ± standard error of the mean (SEM). Statistical analyses were conducted using Minitab Statistical Software (Minitab LLC, State College, PA, USA). One‐way ANOVA followed by Tukey's post hoc test was used to determine statistical differences among groups. Values of *p* < 0.05 were considered statistically significant. FTIR spectra were processed and plotted using Origin software (OriginLab Corporation, Northampton, MA, USA).

## 3 Results and Discussion

### 3.1 Green Synthesis and Characterization of AgNPs

#### 3.1.1 UV‐Vis Spectroscopy

Green synthesis is considered an environmentally friendly and sustainable approach for nanoparticle fabrication owing to the utilization of biological resources acting as reducing and capping agents (Moradi et al. 2021; Eker et al. 2025). Plant‐mediated synthesis offers several advantages, including reduced toxicity, lower energy consumption, improved biocompatibility, and the use of renewable materials (Iravani 2011; Ashtariyan et al. 2024).

**FIGURE 4:**
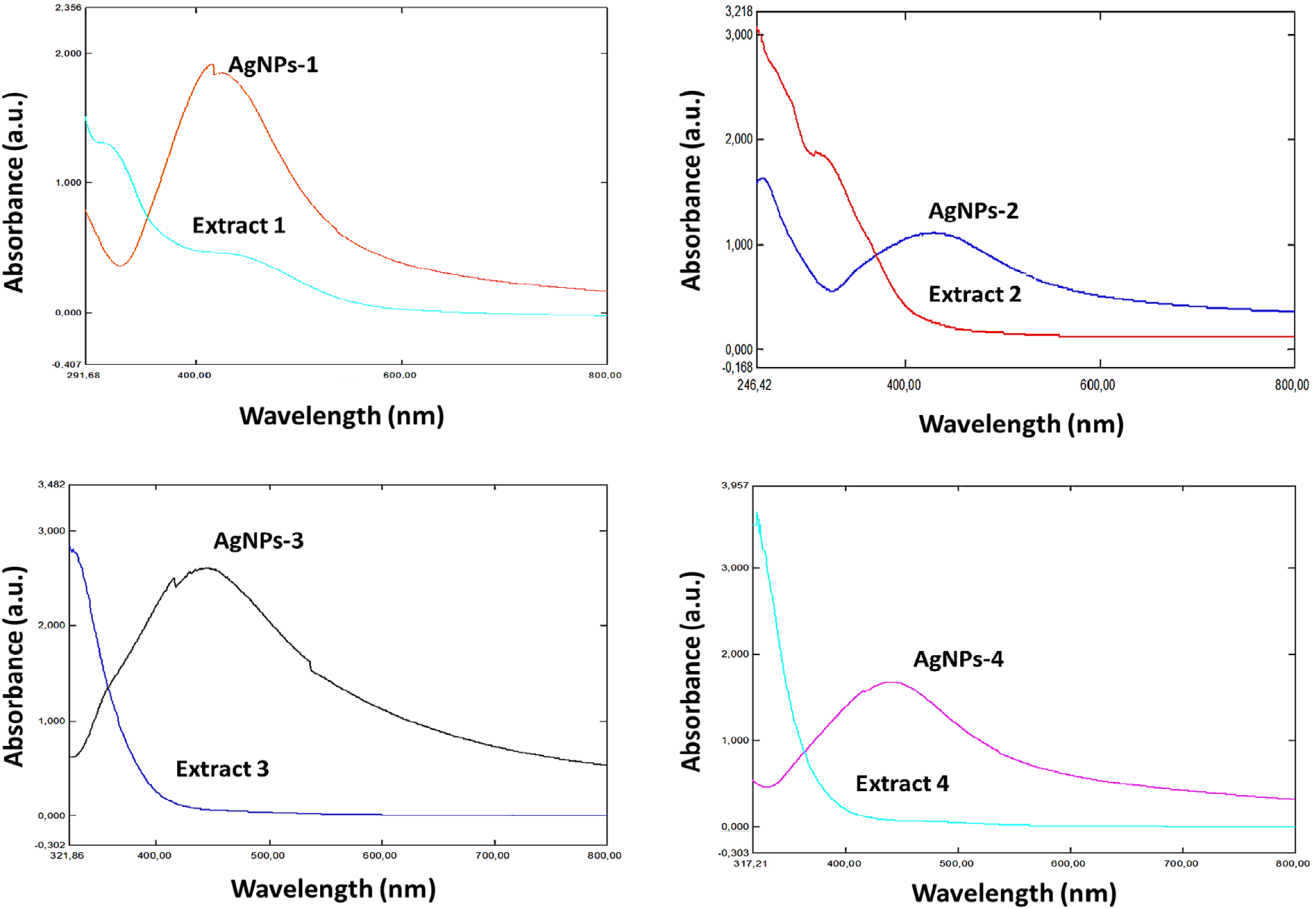
UV‐Vis spectrum of extracts and synthesized AgNPs.

In the present study, the potential application of leaves from four plants, namely, quince ( *C. oblonga* ), kiwi ( *A. deliciosa* ), blackthorn ( *P. spinosa* ), and cherry laurel ( *P. laurocerasus* ), for the synthesis of AgNPs was investigated. UV‐Vis spectroscopy was used to monitor the reaction parameters including extract concentration, AgNO₃ concentration, temperature, and reaction time to achieve a more uniform and narrow size distribution of AgNPs. Initially, nanoparticle synthesis at room temperature resulted in slow reaction rates and non‐uniform nanoparticle formation. Increasing the reaction temperature accelerated nanoparticle formation and improved particle uniformity. Furthermore, dilution of the extracts contributed to a more homogeneous nanoparticle size distribution. The UV‐Vis spectra of the extracts and synthesized AgNPs are shown in Figure 4. The maximum absorption wavelengths (*λ*_max_) for AgNP‐1, AgNP‐2, AgNP‐3, and AgNP‐4 were 423, 430, 445, and 442 nm, respectively. The results obtained are consistent with previous studies and confirm the successful biosynthesis of AgNPs (Baran 2019; Fitriany et al. 2022; Tarighi and Nejad 2023; Bharathi et al. 2024; Hreeba et al. 2025).

#### 3.1.2 Fourier Transform Infrared (FTIR) Spectroscopic Analysis

**FIGURE 5:**
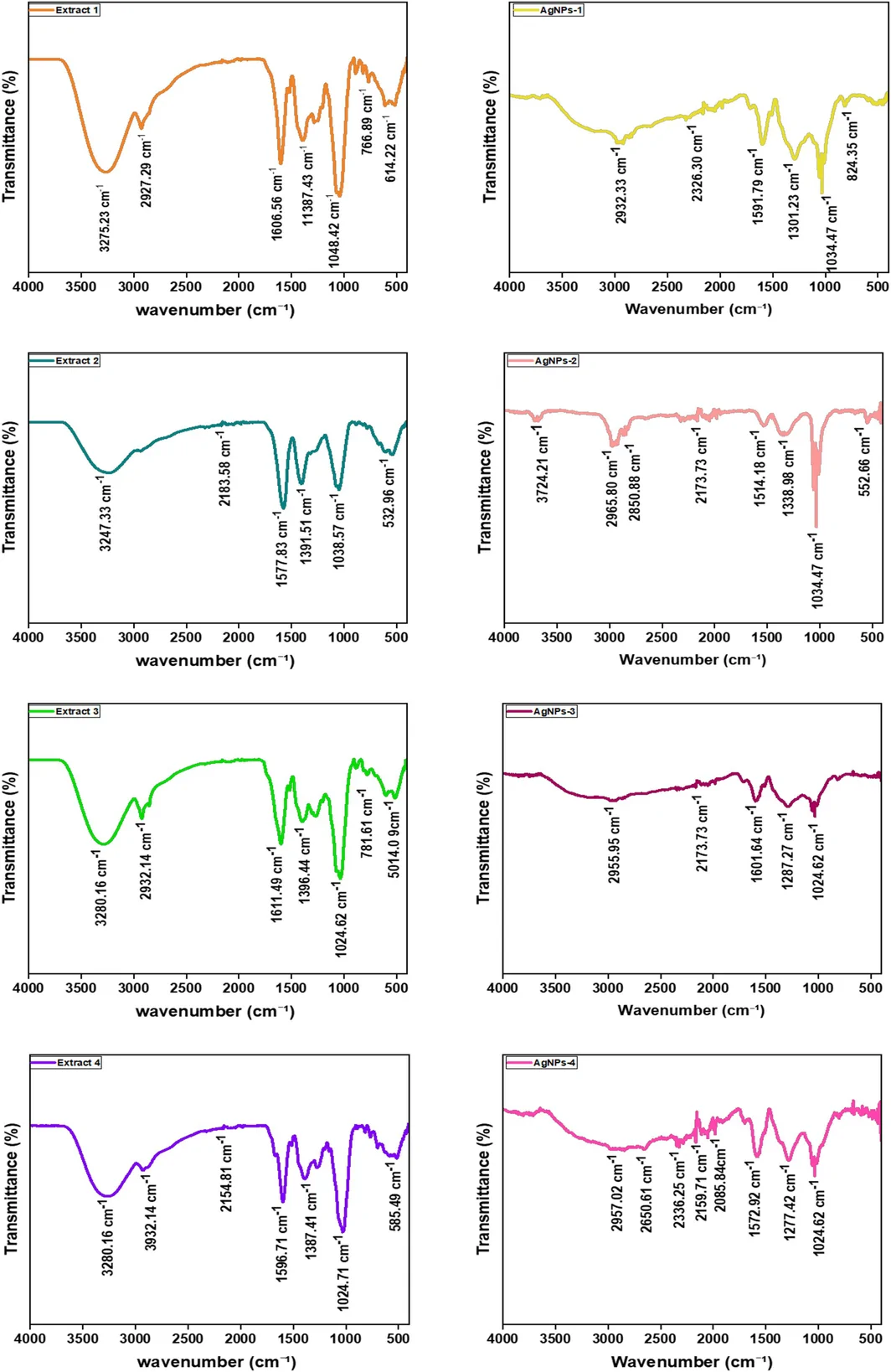
FTIR spectra of extracts and AgNPs.

The FTIR spectra of the plant leaf extracts from *C. oblonga* (Extract‐1), *A. deliciosa* (Extract‐2), *P. spinosa* (Extract‐3), and *P. laurocerasus* (Extract‐4), along with their corresponding biosynthesized silver nanoparticles (AgNPs‐1 to AgNPs‐4), are presented in Figure 5. The obtained spectra confirmed the presence of various functional groups involved in the green synthesis and stabilization of AgNPs. For all analyzed samples, a broad absorption band observed within the range of 3200–3600/cm was attributed to O—H stretching vibrations of phenolic compounds and alcohol groups, indicating the presence of polyphenolic constituents in the extracts (Shukla et al. 2011; Dubey et al. 2017; Aziz et al. 2019). The absorption peaks detected around 2920–2930/cm corresponded to C—H stretching vibrations of aliphatic compounds, whereas the peaks appearing within the 1750–1600/cm region were attributed to C—O stretching vibrations of flavonoids, proteins, and other carbonyl‐containing biomolecules (Sharma et al. 2022; Shantkriti et al. 2023; Aljohani et al. 2026).

Following nanoparticle synthesis, noticeable differences in peak intensities accompanied by slight band shifts were observed between the crude extracts and their corresponding AgNPs. In particular, the O—H stretching bands exhibited reduced intensity and minor shifts after nanoparticle formation, suggesting the involvement of hydroxyl‐containing polyphenols in the reduction of Ag^+^ ions. Similarly, variations within the carbonyl absorption region (1750–1600/cm) indicated possible interactions between silver ions and carbonyl‐containing biomolecules during nanoparticle synthesis. Additional absorption peaks observed within the 1200–1000/cm region were attributed to C—O and C—O—C stretching vibrations of polysaccharides and phenolic compounds. Furthermore, weak absorption bands detected below 600/cm were tentatively attributed to Ag—O bond formation, further supporting the successful biosynthesis of silver nanoparticles (Darwich et al. 2025).

Among the analyzed samples, Extract‐3 and Extract‐4 exhibited comparatively higher absorption intensities in the hydroxyl and carbonyl regions before nanoparticle synthesis, whereas their corresponding AgNPs demonstrated reduced peak intensities after synthesis. In contrast, AgNPs‐2 exhibited relatively weaker spectral features, which may be related to differences in the concentration and composition of the reducing phytochemicals among the investigated plant extracts (Sati et al. 2025). Overall, the observed spectral changes, including peak shifts, intensity reduction, and band broadening, indicate that naturally occurring phytochemicals present in the plant extracts functioned as both reducing and stabilizing agents during the green synthesis of AgNPs (Kirubakaran 2026).

#### 3.1.3 Scanning Electron Microscopy and Energy‐Dispersive X‐Ray Spectroscopic (SEM‐EDX) Analysis

**FIGURE 6:**
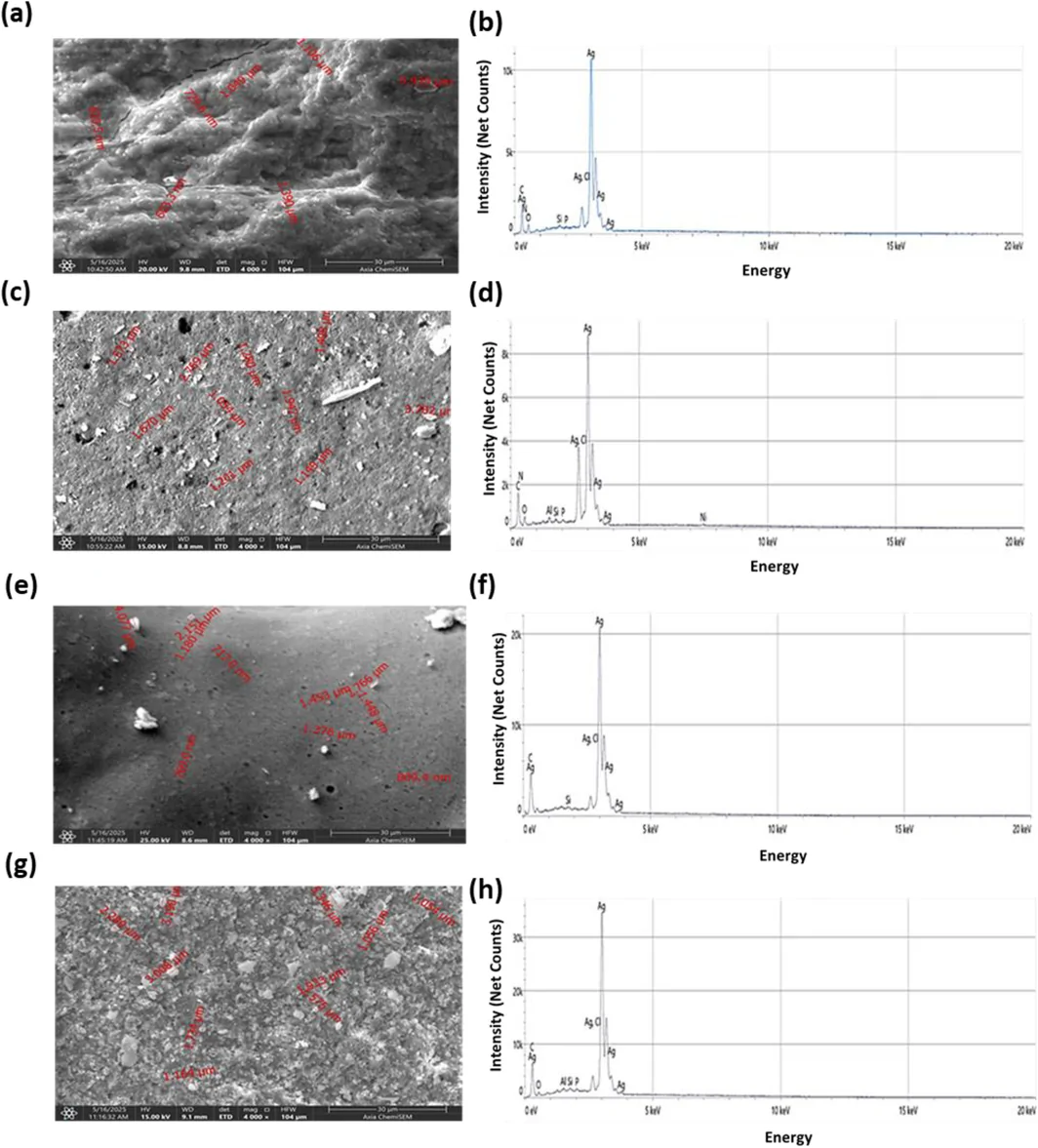
SEM‐EDX analysis of biosynthesized silver nanoparticles (AgNPs‐1–AgNPs‐4). (a, c, e, g) Scanning electron microscopy images; (b, d, f, h) corresponding energy‐dispersive X‐ray spectra showing elemental composition.

SEM‐EDX was used to analyze the morphology and elemental composition of the synthesized silver nanoparticles (AgNPs‐1, AgNPs‐2, AgNPs‐3, and AgNPs‐4) (Figure 6). SEM micrographs revealed noticeable differences in particle morphology, agglomeration behavior, and surface distribution among the synthesized nanoparticles. AgNPs‐1 exhibited a highly irregular and strongly agglomerated surface morphology. In contrast, AgNPs‐2 showed relatively well‐dispersed granular particles distributed across the surface. AgNPs‐3 consisted of comparatively separated particles with a lower agglomeration tendency, whereas AgNPs‐4 demonstrated densely packed granular particles with a more homogeneous surface distribution.

The EDX spectra confirmed the successful formation of the synthesized AgNPs through the presence of characteristic silver peaks around 3.0 keV in all analyzed samples. Silver was detected as the predominant elemental component, indicating the effective reduction of Ag^+^ ions into metallic silver nanoparticles (Darwich et al. 2024). In addition to Ag, minor quantities of O, C, Si, P, Cl, Al, N, and Ni were also detected in some samples. The detection of these elements may be attributed to residual phytochemicals, capping biomolecules originating from the plant extracts, or possible contamination during sample preparation and analysis (Kondak et al. 2024). The elemental composition of the synthesized AgNPs obtained from EDX analysis is summarized in Table 1.

**TABLE 1:** Elemental composition of synthesized AgNP samples determined by EDX analysis.

Sample	Ag (%)	O (%)	C (%)	Si (%)	P (%)	Cl (%)	Al (%)	N (%)	Ni (%)
AgNPs‐1	74.5	9.0	12.3	0.4	0.2	2.2	—	1.4	—
AgNPs‐2	63.1	4.4	20.3	0.3	0.3	8.2	0.6	2.3	0.5
AgNPs‐3	98.4	—	—	0.5	—	1.1	—	—	—
AgNPs‐4	82.7	3.1	11.6	0.4	0.4	1.4	0.4	—	—

Abbreviation: —, not detected.

### 3.2 Phytochemical Characterization, Antioxidant Properties, and Glutathione S‐Transferase (GST) Inhibitory Activity

Phenolic compounds are considered an important group of secondary metabolites, which play a vital role in the biological and antioxidant activity of plants and plant‐derived products. Thus, to assess the phytochemical characteristics of the developed AgNPs, TPC and TFC analyses were conducted. The obtained results are presented in Table 2. Among the synthesized nanoparticles, *P. spinosa* (AgNP‐3) had the highest total phenolic and total flavonoid contents, with 115.63 ± 21.87 mg GAE/g and 112.07 ± 2.87 mg catechin equivalents (CE)/g, respectively, while *A. deliciosa* (AgNP‐2) showed the lowest values, with 12.90 ± 2.76 mg GAE/g and 55.28 ± 3.63 mg CE/g, respectively. In previous literature, TPC and TFC levels for silver nanoparticles synthesized using *Blighia sapida* leaves were 36.52 μg GAE/g and 10.14 μg quercetin equivalents (QE)/g, respectively (Akintola et al. 2020). In another study, the TPC of silver nanoparticles prepared from the extract of *Juniperus procera* was 2.6–6.6 mg GAE/g dry weight, while TFC ranged from 0.75 to 1.0 mg QE/g dry weight (Salih et al. 2022). Likewise, Sivakumar reported TPC and TFC values of 250.14 μg GAE/mg fresh extract and 890.56 μg QE/mg fresh extract, respectively, for AgNPs synthesized using *Cassia auriculata* leaf extracts (Sivakumar 2021). The results generated in the present study are consistent with previous reports on the plant‐based synthesis of silver nanoparticles (Akintola et al. 2020; Sivakumar 2021; Salih et al. 2022). It is well known that phenols and flavonoids exhibit antioxidant properties due to their ability to donate electrons or hydrogen atoms to neutralize reactive oxygen species (ROS) (Niciforovic et al. 2010; Barua et al. 2014; Pang et al. 2018).

**TABLE 2:** Total phenolic and flavonoid contents (TPC, TFC), antioxidant activity (DPPH, ABTS), and GST enzyme inhibitions for silver nanoparticles of *Cydonia oblonga* (AgNP‐1), *Actinidia deliciosa* (AgNP‐2), *Prunus spinosa* (AgNP‐3), and *Prunus laurocerasus* (AgNP‐4).

Sample	TPC (mg GAE/g sample)	TFC (mg catechin/g sample)	IC_50_ for DPPH scavenging (μg/mL)	IC_50_ for ABTS scavenging (μg/mL)	IC_50_ for GST scavenging (mg/mL)
AgNP‐1	47.45 ± 3.43^b^ **	58.86 ± 2.87^c^ **	4.14 ± 0.04^c^ *	29.32 ± 0.17^a^ **	1.31 ± 0.16^b^ **
AgNP‐2	12.90 ± 2.76^c^ **	55.28 ± 3.63^c^ **	10.00 ± 0.20^a,b^ *	22.88 ± 0.32^b^ **	2.24 ± 0.01^a^ **
AgNP‐3	115.63 ± 21.87^a^ **	112.07 ± 2.87^a^ **	7.04 ± 0.25^b,c^ *	29.99 ± 2.01^a^ **	Not active
AgNP‐4	72.45 ± 9.49^b^ **	93.18 ± 2.68^b^ **	12.66 ± 0.12^a^ *	23.15 ± 0.21^b^ **	0.97 ± 0.02^c^ **

*Note:* Data are presented as means of IC_50_ (μg/mL) ± SEM (*n* = 3). ^a^^,^^b^ and ^c^ within the same row indicate statistically significant difference; ^a^^,^^b^ and ^b^^,^^c^ within the same row are not significantly different.

* *p* < 0.05.

** *p* < 0.01.

The antioxidant activity of the synthesized AgNPs was studied using DPPH and ABTS assays, which are commonly employed for evaluating antioxidant activity through radical scavenging activity (Karan, Gonulalan, et al. 2024; Karan, Erenler, et al. 2024; Alam et al. 2025). The green synthesis of AgNPs may enhance antioxidant activity owing to the synergistic effects of AgNPs and the phytochemicals capping the nanoparticles (Liu et al. 2023; Vidya Sagar et al. 2023). The IC_50_ values representing the antioxidant activity of the synthesized AgNPs are given in Table 2. The DPPH IC_50_ values ranged from 4.14 ± 0.04 to 12.66 ± 0.12 μg/mL. Similarly, ABTS IC_50_ values ranged from 22.88 ± 0.32 to 29.99 ± 2.01 μg/mL. Among the four synthesized AgNPs, AgNP‐1, which was synthesized using the leaf extract of *C. oblonga* , exhibited the strongest DPPH scavenging activity (IC_50_ = 4.14 ± 0.04 μg/mL), while AgNP‐4, which was synthesized using the leaf extract of *P. laurocerasus* , showed the weakest activity (IC_50_ = 12.66 ± 0.12 μg/mL). However, for ABTS antioxidant activity, AgNP‐1 and AgNP‐3 showed comparable activities, with IC_50_ values of 29.32 ± 0.17 and 29.99 ± 2.01 μg/mL, respectively.

The enhanced antioxidant activity observed for some AgNPs may be attributed to the synergistic interaction between silver nanoparticles and phytochemicals adsorbed on the nanoparticle surface (Kirubakaran 2025). Phenolic and flavonoid compounds acting as stabilizing agents may contribute significantly to free radical scavenging due to their electron‐donating ability. Similar findings have been reported previously. When *Sambucus ebulus* leaf extract was used for AgNP synthesis, IC_50_ values of 12.1 and 3.1 μg/mL were recorded for DPPH and ABTS scavenging, respectively (Karan, Gonulalan, et al. 2024; Karan, Erenler, et al. 2024). In another study, the IC_50_ values for DPPH and ABTS radical scavenging were 51.80 and 30.78 μg/mL, respectively (Badmus et al. 2020). The IC_50_ values of the AgNPs synthesized from the leaves of *Tagetes erecta* were 23.8 μg/mL for DPPH and 4.5 μg/mL for ABTS (Erenler et al. 2021), while the IC_50_ values of the AgNPs synthesized from *Salvia aethiopis* leaves were 24.37 μg/mL for DPPH and 4.93 μg/mL for ABTS (Gecer 2021). The antioxidant potential of plant‐mediated AgNPs has been extensively investigated using DPPH and ABTS assays. Earlier studies have shown that plant‐mediated AgNPs exhibit stronger radical scavenging activity than the corresponding crude plant extracts, as reflected by lower IC_50_ values (Erenler and Dag 2022). AgNPs have shown the ability to act as antioxidants depending on their concentration, high surface area‐to‐volume ratio, and the presence of bioactive phytochemicals acting as capping agents (Zare‐Bidaki et al. 2023). In addition, Krishnamoorthy et al. (2023) reported that AgNPs synthesized from *Argyreia nervosa* leaf extract exhibited strong DPPH, ABTS, and H_2_O_2_ scavenging abilities due to the interaction between the AgNPs and the phytochemicals. Moreover, AgNPs obtained from *Salvia officinalis* and *Thymus vulgaris* showed strong DPPH scavenging activity (Odemis et al. 2022).

Glutathione S‐transferase (GST) is an important detoxification enzyme that plays an essential role in cellular defense mechanisms (Fakae et al. 2000). Elevated activity of GST has been observed in many diseased states, including cancer; thus, inhibition of GST has been proposed as a therapeutic target (Kandemir et al. 2024; Sahın and Sonmez 2025). The IC_50_ values for GST inhibition by the synthesized nanoparticles ranged from 0.97 ± 0.02 to 2.24 ± 0.01 mg/mL. AgNP‐4, which was synthesized using the leaf extract of *P. laurocerasus* , showed the highest GST inhibitory activity (IC_50_ = 0.97 ± 0.02 mg/mL), while AgNP‐3 showed no significant inhibitory activity (Table 2). Previous studies have shown that phytochemicals acting as capping agents on the surface of nanomaterials play a major role in determining their biological activities. According to the findings of Yadav et al., green‐synthesized AgNPs obtained from *Syzygium aromaticum* demonstrated greater antioxidant capacity than chemically synthesized AgNPs because of the presence of phytochemical capping agents (Yadav and Preet 2023). Fakae et al. (2000) studied the inhibitory activity of GST using various medicinal plant extracts and concluded that the IC_50_ values ranged between 2 and 28 mg/mL, while the IC_50_ value for *Rumex acetosella* extract was 9.10 mg/mL (Kandemir et al. 2024). Moreover, AgNPs synthesized using *Azadirachta indica* and *Althaea officinalis* have been reported to modulate GST‐related biochemical processes (Rheder et al. 2018; Bhat et al. 2019).

### 3.3 Anti‐Inflammatory Activity

Inflammation is a protective biological response triggered by harmful stimuli such as pathogens, toxins, or tissue injury. However, chronic and uncontrolled inflammatory responses are associated with the development of several diseases, including rheumatoid arthritis, atherosclerosis, chronic liver disorders, and cancer (Hamid and Salih 2022; Nunes et al. 2022). Protein denaturation is considered one of the major mechanisms involved in inflammatory processes. Therefore, inhibition of protein denaturation is commonly used as an in vitro indicator of anti‐inflammatory potential in preclinical studies (Banerjee et al. 2014; Derbel et al. 2023).

**FIGURE 7:**
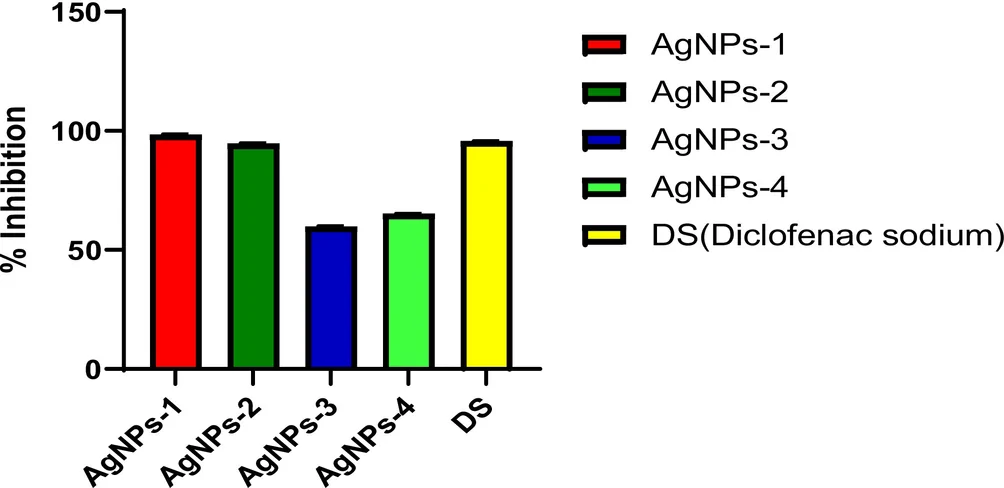
Anti‐inflammatory activity of biosynthesized AgNPs and diclofenac sodium measured by the protein denaturation inhibition assay.

Figure 7 shows the percentage inhibition values of the anti‐inflammatory activity of the synthesized nanoparticles (AgNPs‐1–4) and the positive control, diclofenac sodium, which was used as the reference anti‐inflammatory drug. The percentage inhibition values obtained for AgNP‐1, AgNP‐2, AgNP‐3, and AgNP‐4 were 98.48%, 94.69%, 59.84%, and 65.15%, respectively. AgNP‐1 showed stronger anti‐inflammatory effects than the positive control, diclofenac sodium (95.61% inhibition), whereas AgNP‐2 exhibited comparable anti‐inflammatory activity. On the other hand, AgNP‐3 and AgNP‐4 showed comparatively lower inhibition values than diclofenac sodium.

Kanimozhi reported that the inhibition percentages of BSA denaturation by silver nanoparticles ranged from 25% to 81%, depending on nanoparticle concentration (Kanimozhi et al. 2022). Another study reported that silver nanoparticles exhibited anti‐inflammatory activity with inhibition percentages varying from 50% to 89% when assessed using the protein denaturation method (Sabarathinam and Madhulaxmi 2021). Mohamed and colleagues synthesized tenoxicam/meloxicam‐loaded silver nanoparticles using different plant extracts and reported inhibition percentages ranging between 7.66% and 62.57% (Mohamed et al. 2024). The strong anti‐inflammatory activity observed for AgNP‐1 and AgNP‐2 in the present study may be associated with the synergistic effects between silver nanoparticles and phytochemical capping agents, particularly polyphenols and flavonoids known for their anti‐inflammatory properties. The anti‐inflammatory properties of AgNPs‐1–4 synthesized from *C. oblonga* , *A. deliciosa* , *P. spinosa* , and *P. laurocerasus* leaves in the present study were comparable to or stronger than those reported in previous studies.

### 3.4 Evaluation of Antibacterial Activity

#### 3.4.1 Disk Diffusion Assay and Inhibition Zone Analysis

The increasing prevalence of antibiotic‐resistant bacteria has intensified the search for alternative antimicrobial agents. Green‐synthesized silver nanoparticles have attracted considerable attention due to their broad‐spectrum antibacterial activity, high surface area‐to‐volume ratio, and ability to interact with bacterial membranes and induce oxidative stress (Cowan 1999; Sharifi et al. 2012). Phytochemical‐functionalized AgNPs may exhibit enhanced antibacterial activity due to the synergistic interactions between silver nanoparticles and plant‐derived bioactive compounds (Dai et al. 2020).

**FIGURE 8:**
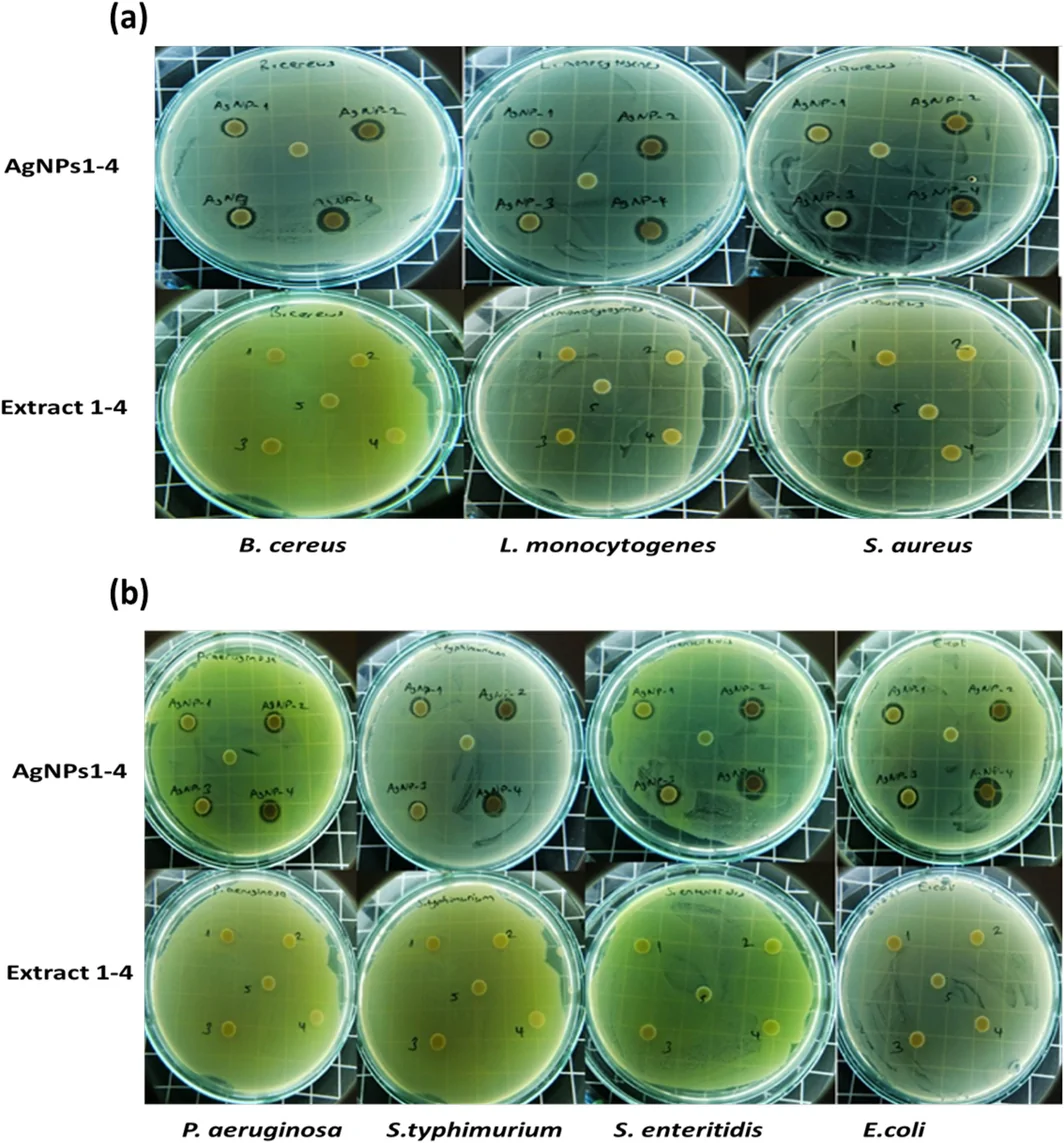
Representative agar well diffusion images showing the antibacterial activity of synthesized AgNPs against (a) Gram‐positive and (b) Gram‐negative bacterial strains.

The antibacterial activity of silver nanoparticles (AgNPs‐1–4) synthesized from the studied species was evaluated in vitro using the disk diffusion and minimum inhibitory concentration (MIC) methods. The crude extracts did not exhibit inhibition zones against the tested bacterial strains, while all AgNPs showed inhibitory activity against all investigated bacteria. The inhibition zones are presented in Table 3 and Figure 8. The inhibition zone diameters ranged from 8.35 ± 0.17 to 10.25 ± 0.08 mm against *P. aeruginosa* , from 8.26 ± 0.06 to 10.72 ± 0.64 mm against *S. typhimurium* , from 8.86 ± 0.34 to 10.97 ± 0.47 mm against *S. enteritidis* , from 9.03 ± 0.32 to 11.22 ± 0.29 mm against *E. coli* , from 8.59 ± 0.15 to 10.22 ± 0.06 mm against *B. cereus* , from 8.42 ± 0.10 to 10.24 ± 0.05 mm against *L. monocytogenes* , and from 8.86 ± 0.48 to 10.44 ± 0.19 mm against *S. aureus* . The inhibition zones of ampicillin, used as a positive control, were determined as 20.92 ± 0.01 mm against *P. aeruginosa* , 21.15 ± 0.99 mm against *S. typhimurium* , 22.10 ± 1.41 mm against *S. enteritidis* , 16.17 ± 0.25 mm against *E. coli* , 18.09 ± 1.07 mm against *B. cereus* , 17.29 ± 0.26 mm against *L. monocytogenes* , and 19.08 ± 0.88 mm against *S. aureus* . These values were significantly higher than those observed for the synthesized AgNPs, indicating the greater antibacterial efficacy of the standard antibiotic under the experimental conditions. However, although silver nanoparticles have demonstrated antibacterial activity in the literature, limitations such as dose optimization and uncertainties regarding their mechanism of action suggest that they are more appropriately considered supportive or complementary antimicrobial agents capable of enhancing the effectiveness of conventional antibiotics when used in combination, rather than direct replacements for classical antibiotics in clinical practice (More et al. 2023).

**TABLE 3:** Diameters of inhibition zones (mm) of synthesized AgNPs against tested bacteria.

Bacterial strains	Diameters of inhibition zones (mm)			
	AgNP‐1	AgNP‐2	AgNP‐3	AgNP‐4
Gram negatives				
*Pseudomonas aeruginosa*	8.95 ± 0.22^b^**^,A^	9.98 ± 0.19^a^**^,A^	8.35 ± 0.17^b^**^,A^	10.25 ± 0.08^a^**^,A^
*Salmonella typhimurium*	8.55 ± 0.09^b^**^,A^	10.12 ± 0.10^a^**^,A^	8.26 ± 0.06^b^**^,A^	10.72 ± 0.64^a^**^,A^
*Salmonella enteritidis*	9.29 ± 0.20^b,c^**^,A^	10.15 ± 0.28^a,b^**^,A^	8.86 ± 0.34^c^**^,A^	10.97 ± 0.47^a^**^,A^
*Escherichia coli*	9.14 ± 0.51^b^**^,A^	10.33 ± 0.50^a,b^**^,A^	9.03 ± 0.32^b^**^,A^	11.22 ± 0.29^a^**^,A^
Gram positives				
*Bacillus cereus*	9.26 ± 0.26^b^**^,A^	10.14 ± 0.32^a^**^,A^	8.59 ± 0.15^b^**^,A^	10.22 ± 0.06^a^**^,A^
*Listeria monocytogenes*	8.45 ± 0.09^b^**^,A^	9.93 ± 0.24^a^**^,A^	8.42 ± 0.10^b^**^,A^	10.24 ± 0.05^a^**^,A^
*Staphylococcus aureus*	8.88 ± 0.07^b^*^,A^	10.44 ± 0.64^a^*^,A^	8.86 ± 0.48^b^*^,A^	10.44 ± 0.19^a^*^,A^

*Note:* Values were presented as mean ± SD, obtained from three separate sets of experiments (*n* = 3). Different lowercase letters in the same row denote the statistically significant difference, whereas different uppercase letters in the same column denote the significant difference between various groups. *p < 0.05 and **p < 0.01.

In a study conducted by Liaqat et al. (2022), inhibition zone values of green‐synthesized AgNPs against *S. aureus* and *E. coli* ranged from 11 to 17 mm and from 7 to 12 mm, respectively. Similarly, AgNPs synthesized using *Oxalis griffithii* extract exhibited an inhibition zone diameter of 10 ± 0.91 mm against *E. coli* (Singla et al. 2022). Sangaonkar and Pawar (2018) synthesized AgNPs using *Garcinia indica* fruit extract and reported inhibition zone values ranging from 12 ± 0.5 to 15 ± 0.2 mm against *E. coli* , *S. aureus* , and *P. aeruginosa* , whereas *S. enteritidis* was not inhibited. Tolouietabar et al. (2020) also reported significant antibacterial activity of AgNPs synthesized using *Scrophularia striata* extract against *S. aureus* , *B. cereus* , *E. coli* , and *S. typhimurium* . Overall, the antibacterial activities observed for AgNP‐2 and AgNP‐4, synthesized from *A. deliciosa* and *P. laurocerasus* leaf extracts, were comparable to or stronger than those of several previously reported plant‐mediated AgNPs, highlighting their promising antibacterial potential.

#### 3.4.2 Determination of MIC and MBC Profiles of the Synthesized Samples Toward the Selected Bacterial Isolates

The minimum inhibitory concentration (MIC) and minimum bactericidal concentration (MBC) values for the prepared AgNPs were determined against three Gram‐positive bacteria ( *B. cereus* , *L. monocytogenes* , and *S. aureus* ) and four Gram‐negative bacteria ( *P. aeruginosa* , *S. typhimurium* , *S. enteritidis* , and *E. coli* ) (Table 4). There was no inhibitory or bactericidal activity observed for the crude extracts against any of the bacterial strains tested. Conversely, the synthesized AgNPs exhibited strong inhibitory and bactericidal activities against all Gram‐positive and Gram‐negative bacteria.

**TABLE 4:** Minimum inhibitory concentration (MIC) and minimum bactericidal concentration (MBC) values of the synthesized AgNPs against the tested bacterial strains.

Bacterial strains	MIC (μg/mL)				MBC (μg/mL)			
	AgNP‐1	AgNP‐2	AgNP‐3	AgNP‐4	AgNP‐1	AgNP‐2	AgNP‐3	AgNP‐4
Gram negatives								
*Pseudomonas aeruginosa*	156	78	313	39	625	156	625	78
*Salmonella typhimurium*	313	78	313	39	625	313	625	78
*Salmonella enteritidis*	156	39	313	39	625	156	625	78
*Escherichia coli*	156	78	313	39	1250	313	1250	156
Gram positives								
*Bacillus cereus*	313	39	313	39	313	313	1250	78
*Listeria monocytogenes*	156	78	625	78	313	156	625	156
*Staphylococcus aureus*	313	78	313	39	5000	1250	5000	2500

Abbreviations: AgNPs, silver nanoparticles; MBC, minimum bactericidal concentration; MIC, minimum inhibitory concentration.

The minimum inhibitory concentration (MIC) values ranged from 39 to 313 μg/mL against most of the tested bacterial strains, whereas *L. monocytogenes* exhibited MIC values ranging from 78 to 625 μg/mL. The minimum bactericidal concentration (MBC) values ranged from 78 to 625 μg/mL against *P. aeruginosa* , from 78 to 625 μg/mL against *S. typhimurium* , from 78 to 625 μg/mL against *S. enteritidis* , from 156 to 1250 μg/mL against *E. coli* , from 78 to 1250 μg/mL against *B. cereus* , from 156 to 625 μg/mL against *L. monocytogenes* , and from 1250 to 5000 μg/mL against *S. aureus* . Overall, the MIC and MBC values of the synthesized AgNPs ranged from 39 to 625 μg/mL and from 78 to 5000 μg/mL, respectively.

The highest bacteriostatic and bactericidal efficacy was achieved with AgNP‐4, as it showed the strongest antibacterial activity against both Gram‐positive and Gram‐negative bacteria. AgNP‐3 was the least effective formulation. AgNP‐2 exhibited a bacteriostatic effect comparable to that of AgNP‐4 and greater than those of AgNP‐1 and AgNP‐3. Regarding bactericidal activity, the most susceptible bacterial strains varied depending on the synthesized AgNP formulation. AgNP‐4 demonstrated the strongest bactericidal activity, particularly against *P. aeruginosa* , *S. typhimurium* , *S. enteritidis* , and *B. cereus* , with MBC values as low as 78 μg/mL. Among the investigated microorganisms, *S. aureus* exhibited the highest resistance to all synthesized AgNPs.

Previous studies indicated that silver nanoparticles synthesized using *Scrophularia striata* extract showed antibacterial activity, with MIC values ranging from 156 μg/mL against *S. typhi* , *S. aureus* , and *B. cereus* to 312 μg/mL against *E. coli* . Similarly, the MBC values ranged from 312 μg/mL against *S. typhi* , *S. aureus* , and *B. cereus* to 625 μg/mL against *E. coli* (Tolouietabar et al. 2020). Another report demonstrated that silver nanoparticles biosynthesized using the aqueous tuber extract of *Gloriosa superba* exhibited antibacterial efficacy against *Enterococcus faecalis* , *Bacillus subtilis* , and *S. aureus* (Murugesan et al. 2021). In the study performed by Odemis and colleagues, AgNPs synthesized using *Salvia officinalis* and *Thymus vulgaris* extracts demonstrated antimicrobial activity against bacteria and fungi. The MIC values of SO‐AgNPs and TV‐AgNPs ranged from 32 to 256 mg/L and from 64 to 512 mg/L, respectively (Odemis et al. 2022). Overall, the antibacterial findings of the present study, particularly for AgNP‐4 and AgNP‐2 synthesized from *P. laurocerasus* and *A. deliciosa* leaf extracts, were comparable to or, in some cases, stronger than those reported in several previous studies, highlighting their promising antibacterial potential.

Antibacterial and GST inhibition analyses revealed that AgNPs‐2 and AgNPs‐4 exhibited the highest activity, as reflected by their lowest MIC and MBC values. In contrast, AgNPs‐3 (98.4% Ag), which had the highest silver content, showed lower antibacterial activity. This indicates that the antibacterial activity is not solely dependent on the amount of silver. Aggregation and environmental organic/inorganic components can influence Ag^+^ release kinetics and dissolution behavior through particle aggregation and complex formation (Shanmuganathan et al. 2018). SEM results showed a more homogeneous distribution for AgNPs‐2 and AgNPs‐4, while AgNPs‐1 and AgNPs‐3 showed significant aggregation and a more heterogeneous structure. It is well known that a homogeneous structure and a higher surface area enhance bacterial contact and Ag^+^ release, thereby strengthening antibacterial activity (Dakal et al. 2016). EDX analyses revealed the presence of C, O, and N on the surface of AgNPs‐2 and AgNPs‐4, suggesting the stabilizing/capping role of plant‐derived biomolecules. This organic coating can enhance nanoparticle stability and Ag^+^ bioavailability (Ahmed et al. 2016). In general, antibacterial and GST inhibitory activities are primarily influenced by nanoparticle morphology, aggregation state, and surface functionalization rather than silver co.

### 3.5 Molecular Docking

**FIGURE 9:**
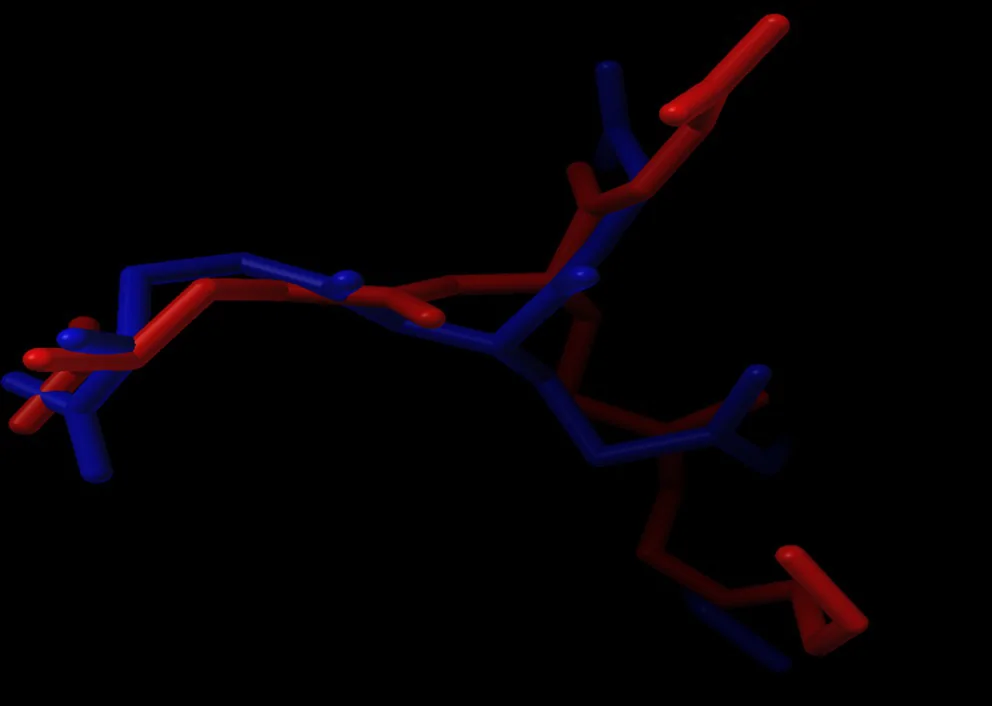
Comparison of the native glutathione adduct of hexyl‐isothiocyanate ligand (red) and re‐docked co‐crystallized ligand (blue) binding orientations by superposition.

Computational docking analysis was conducted to examine the interaction mechanisms among the selected phytochemical constituents, AgNPs, and the active‐site residues of the glutathione S‐transferase (GST) enzyme (Morris et al. 2009; Kadir et al. 2024). In this study, the reliability of the selected docking parameters was assessed by calculating the root‐mean‐square deviation (RMSD) between the native glutathione adduct of the hexyl‐isothiocyanate ligand (red) and the re‐docked co‐crystallized ligand (blue) based on their structural superposition, as illustrated in Figure 9. The resulting RMSD value of 1.48 Å demonstrates that the docking protocol was valid and appropriate. Consequently, the optimized parameters were employed for docking the selected phytochemicals and AgNPs into the active site of the GST structure.

**FIGURE 10:**
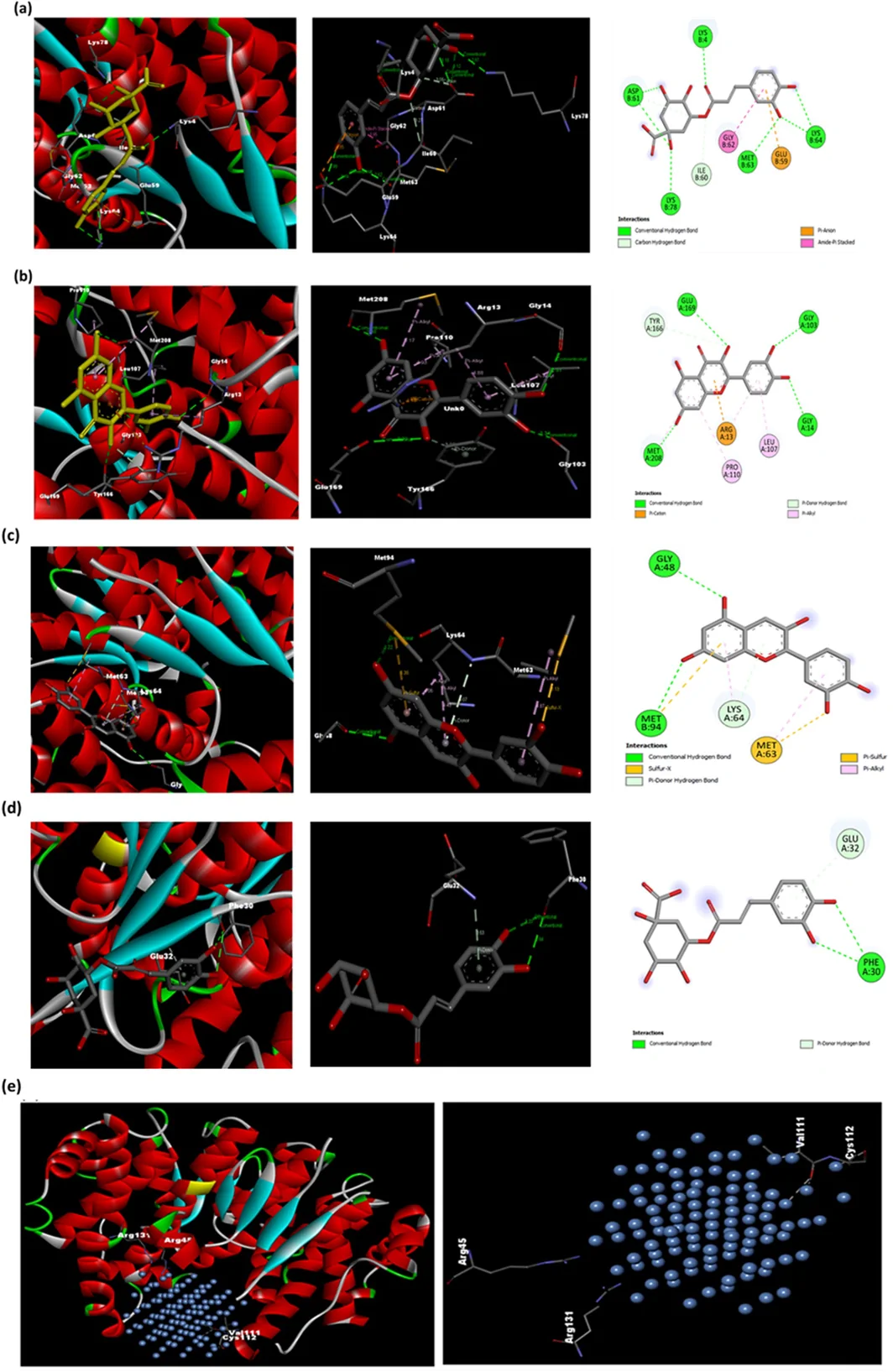
Two‐dimensional and three‐dimensional visual representations of the molecular interactions among the major compounds identified in Extracts 1–4, AgNPs, and GST. Interactions of (a‐d) Extracts 1‐4 and (e) AgNPs with GST.

Based on the molecular docking analysis, the interactions between the protein and the ligands, along with their corresponding interaction distances, are summarized in Table 5. Moreover, Figure 10 shows both two‐dimensional and three‐dimensional representations of the binding interactions between the selected compounds and the active‐site residues of GST. Molecular docking results showed that the aromatic hydroxyl groups of the phytochemical constituents found in the extracts formed interaction bonds with amino acid residues such as LYS, MET, ASP, GLY, GLU, TYR, and PHE located within the active site of GST through conventional hydrogen bonds and/or π‐donor hydrogen bond formation. Furthermore, electrostatic and hydrophobic interactions, including π–anion, π–cation, amide–π stacked, π–alkyl, and π–sulfur interactions, were observed between the aromatic ring structures of the phytochemical constituents and the GLU, GLY, TYR, PRO, MET, ARG, LEU, and LYS amino acid residues. Ag atoms in the nanoparticles interacted with VAL and CYS residues of the enzyme through metal–acceptor interactions.

**TABLE 5:** Binding interaction profiles and interaction distance values of the principal phytochemical compounds from Extracts 1 to 4 and AgNPs against the target protein based on molecular docking results.

Substrate	Receptor	Interaction	Distance (Å)	*K*_i_ (mM)	Binding energy (kcal/mol)
Extract 1 (5‐*O*‐caffeoylquinic acid)					
C=O (O)	LYS4 (N)	Conventional H‐bond	2.75	1.23	−3.97
Ph–OH	LYS64 (NZ)	Conventional H‐bond	3.19		
Ph–OH	LYS64 (NZ)	Conventional H‐bond	2.99		
Caffeoyl–OH	LYS78 (NZ)	Conventional H‐bond	2.87		
Caffeoyl–OH	MET63 (O)	Conventional H‐bond	2.62		
Caffeoyl–OH	ASP61 (OD1)	Conventional H‐bond	3.12		
Caffeoyl–OH	ASP61 (OD1)	Conventional H‐bond	3.18		
*O*‐CO (O)	ILE60 (CA)	Carbon H‐bond	3.27		
Caffeoyl carbon	ASP61 (OD1)	Carbon H‐bond	3.08		
Aromatic phenyl ring	GLU59 (OE2)	Electrostatic (pi‐anion)	4.35		
Aromatic phenyl ring	GLY62 (C, O)	Hydrophobic (amide‐pi stacked)	3.95		
Extract 2 (quercetin)					
Ph–OH	GLY103 (O)	Conventional H‐bond	3.34	0.79	−4.23
Ph–OH	GLY14 (O)	Conventional H‐bond	2.91		
Pyranone–OH	GLU169 (OE2)	Conventional H‐bond	3.30		
Benzopyranone–OH	MET208 (O)	Conventional H‐bond	2.98		
Pyranone	ARG13 (NH1)	Electrostatic (pi‐cation)	4.10		
Pyranone–OH	TYR166	Pi‐donor H‐bond	3.83		
Benzopyranone	PRO110	Hydrophobic (pi‐alkyl)	4.93		
Benzopyranone	MET208	Hydrophobic (pi‐alkyl)	5.17		
Aromatic phenyl ring	ARG13	Hydrophobic (pi‐alkyl)	4.88		
Aromatic phenyl ring	LEU107	Hydrophobic (pi‐alkyl)	4.76		
Extract 3 (cyanidin)					
Benzopyran–OH	GLY48 (O)	Conventional H‐bond	2.69	3.29	−3.39
Benzopyran–OH	MET94 (SD)	Conventional H‐bond	3.22		
Aromatic phenyl ring	MET63 (SD)	Sulfur‐X	3.13		
Pyran‐O	LYS64 (N)	Pi‐donor H‐bond	4.07		
Benzopyran	MET94 (SD)	Pi‐sulfur	5.36		
Benzopyran	LYS64	Hydrophobic (pi‐alkyl)	4.06		
Pyran	LYS64	Hydrophobic (pi‐alkyl)	4.43		
Aromatic phenyl ring	MET63	Hydrophobic (pi‐alkyl)	4.87		
Extract 4 (chlorogenic acid)					
Ph–OH	PHE30 (O)	Conventional H‐bond	3.20	3.35	−3.38
Ph–OH	PHE30 (O)	Conventional H‐bond	2.64		
Aromatic phenyl ring	GLU32 (N)	Pi‐donor H‐bond	3.63		
AgNP					
Ag	VAL111 (O)	Metal‐acceptor	3.16		
Ag	VAL111 (O)	Metal‐acceptor	3.38		
Ag	CYS112 (O)	Metal‐acceptor	3.06		

*Note:* Hydrogen bonding, hydrophobic, electrostatic, and metal–acceptor interactions were identified through molecular docking analysis.

Abbreviations: Å, angstrom; AgNP, silver nanoparticle; C=O, carbonyl group; ph–OH, phenolic hydroxyl group; pyran‐O, pyran oxygen; pi‐alkyl, π–alkyl interaction; pi‐anion, π–anion interaction; pi‐cation, π–cation interaction.

Aras and collaborators reported GST enzyme inhibition activity of the endemic plant species *Thymus migricus*, which contains several phenolic acid compounds, namely quinic acid, chlorogenic acid, and *p*‐coumaric acid (Aras et al. 2021). Molecular docking analysis in their study revealed that conventional hydrogen bonds and carbon–hydrogen bonds were the predominant interactions between phenolic compounds and GLN, VAL, SER, GLU, ARG, and ASP residues of GST. Ozcan and collaborators investigated the interaction between GST and quercetin using molecular docking analysis and observed hydrogen bonding, π–π stacking, and van der Waals interactions involving SER14, TYR7, LYS44, HIS49, and ALA12 residues within the enzyme active site (Ozcan et al. 2024). Similarly, anthocyanins, including cyanidin derivatives from *Rosa chinensis* , demonstrated strong interactions with GST amino acid residues through multiple hydrogen bonds and van der Waals interactions (Zhang et al. 2025). Additionally, Abd Kadir and collaborators demonstrated that amino acid residues within protein pockets could interact directly with silver atoms through metal‐related and nonbonding interactions on the surface of AgNPs (Kadir et al. 2024). A limitation of the molecular docking analysis is that AgNPs were represented by a simplified silver atom model rather than the complete nanoparticle structure. Due to the computational limitations associated with modeling large and heterogeneous nanoparticle systems, the docking approach was intended only to provide preliminary insights into possible metal–protein interactions. The selected phytochemicals were chosen as representative major bioactive components reported as dominant compounds in the relevant plant extracts according to previously published phytochemical profiling studies. Another limitation of this study concerns the molecular docking approach. We performed docking studies using representative selected phytochemicals and a simplified Ag model and do not fully represent the complexity of phytochemical‐coated AgNPs. Moreover, the docking calculations provide only static interaction predictions and do not consider solvent effects, protein flexibility, the heterogeneity of the nanoparticle surface, and dynamic biological environments. Therefore, the docking results should be regarded as supportive mechanistic evidence rather than conclusive evidence for the inhibition of GST. Overall, these findings support the molecular docking results obtained in the present study and suggest that phytochemical constituents together with AgNPs may contribute to GST inhibitory activity through multiple molecular interaction mechanisms.

## 4 Conclusion

The successful green synthesis of silver nanoparticles (AgNPs) was achieved using leaf extracts of *C. oblonga* , *A. deliciosa* , *P. spinosa* , and *P. laurocerasus* . The synthesized AgNPs demonstrated promising biological activities, including antioxidant, antibacterial, anti‐inflammatory, and glutathione S‐transferase (GST) inhibitory properties. All synthesized AgNPs exhibited considerable antioxidant potential through effective scavenging of DPPH and ABTS radicals. In addition, AgNP‐1, AgNP‐2, and AgNP‐4 showed notable GST inhibitory activity. Among the investigated nanoparticles, AgNP‐2 and AgNP‐4 demonstrated comparatively stronger antibacterial activity against both Gram‐positive and Gram‐negative bacterial strains, whereas AgNP‐1 exhibited remarkable anti‐inflammatory activity. Overall, the obtained findings demonstrate the promising in vitro antioxidant, antibacterial, anti‐inflammatory, and GST inhibitory activities of plant‐mediated AgNPs. The observed biological activities may be associated with the synergistic interactions between silver nanoparticles and phytochemical capping agents present on the nanoparticle surface. However, additional mechanistic studies, cytotoxicity evaluations, and in vivo investigations are required to validate these findings and assess their potential biomedical applications.

One limitation of the present study is the absence of advanced physicochemical characterization techniques such as X‐ray diffraction (XRD), dynamic light scattering (DLS), zeta potential analysis, and transmission electron microscopy (TEM), which could provide additional information regarding the crystalline structure, particle size distribution, colloidal stability, and detailed morphology of the synthesized nanoparticles. Therefore, future studies should incorporate these characterization methods to achieve a more comprehensive understanding of nanoparticle structure and stability. Future investigations should focus on the in vivo evaluation of the synthesized AgNPs to assess their long‐term safety, pharmacokinetic behavior, and toxicological profiles. In addition, further molecular and mechanistic studies are required to better understand the interactions of AgNPs within biological systems, particularly regarding GST inhibition mechanisms. Optimization of large‐scale synthesis conditions and the assessment of the reproducibility of nanoparticle production will also be essential for future biomedical and pharmaceutical applications. Furthermore, the incorporation of green‐synthesized AgNPs into nanostructured drug delivery systems or combination therapies may offer promising opportunities in nanomedicine.

## Author Contributions

**Zuhal Sahin:** investigation, conceptualization, methodology, data curation, formal analysis, validation, writing – original draft, writing – review and editing. **Nourhane A. Darwich:** investigation, conceptualization, validation, visualization, methodology, data curation, formal analysis, literature search, software, supervision, writing – original draft, writing – review and editing. **Tulay Duran:** conceptualization, methodology, formal analysis, validation. **Davut Avci:** formal analysis, validation, software, writing – original draft. **Luís R. Silva:** writing – original draft, writing – review and editing. **Fatih Sonmez:** conceptualization, methodology, supervision, writing – original draft, writing – review and editing. **Mahmoud I. Khalil:** conceptualization, validation, visualization, methodology, supervision, writing – review and editing.

## Funding

This work was supported by Sakarya University of Applied Sciences (164‐2023).

## Ethics Statement

This study did not involve human participants, human data, human tissues, or animals. Therefore, ethical approval and informed consent were not required.

## Conflicts of Interest

The authors declare no conflicts of interest.

## Data Availability

All the relevant information regarding this study is included in this manuscript. If additional datasets are needed for future experiments, they are available from the corresponding author upon appropriate request.
